# A new two-decade (2001–2019) high-resolution agricultural primary productivity dataset for India

**DOI:** 10.1038/s41597-022-01828-y

**Published:** 2022-11-27

**Authors:** Prasun K. Gangopadhyay, Paresh B. Shirsath, Vinay K. Dadhwal, Pramod K. Aggarwal

**Affiliations:** 1 Borlaug Institute for South Asia (BISA), International Maize and Wheat Improvement Centre (CIMMYT), New Delhi, India; 2 National Institute of Advanced Studies (NIAS), Bengaluru, Karnataka India

**Keywords:** Agriculture, Governance

## Abstract

The present study describes a new dataset that estimates seasonally integrated agricultural gross primary productivity (GPP). Several models are being used to estimate GPP using remote sensing (RS) for regional and global studies. Using biophysical and climatic variables (MODIS, SBSS, ECWMF reanalysis etc.) and validated by crop statistics, the present study provides a new dataset of agricultural GPP for monsoon and winter seasons in India for two decades (2001–2019). This dataset (GPPCY-IN) is based on the light use efficiency (LUE) principle and applied a dynamic LUE for each year and season to capture the seasonal variations more efficiently. An additional dataset (NGPPCY-IN) is also derived from crop production statistics and RS GPP to translate district-level statistics at the pixel level. Along with validation with crop statistics, the derived dataset was also compared with *in situ* GPP estimations. This dataset will be useful for many applications and has been created for estimating integrated yield loss by taking GPP as a proxy compared to resource and time-consuming field-based methods for crop insurance.

## Abstract (summary)

**Table:** 

Measurement(s)	gross primary productivity
Technology Type(s)	remote sensing
Factor Type(s)	vegetation, radiation, water
Sample Characteristic - Organism	vegetation
Sample Characteristic - Location	India

## Background & Summary

The recent IPCC report^1^ has once again highlighted that current climatic variability and increasing weather risks associated with climate change threaten agricultural production systems and food security all over the world. Such assessments project global crop yields project would continue to decline due to climate change; losses going up to 50% by the 2080 s, especially in low latitudes countries in sub-Saharan Africa and South Asia. Several technological, institutional and policy interventions have been proposed that can help the world to adapt to climate change. However, there is a need to realistically understand the vulnerability and adaptation options in developing countries where the landholding size of most of the farmers is small. In South Asia, for example, 85% of farmers (>150 million farm households) have a landholding size of fewer than 2 hectares. One needs historical yield and weather data to understand trends in impacts and to design relevant and site-specific adaptation interventions such as insurance products. Multiple datasets have been developed to analyze the trends and temporal variation in yields that are based on the Food and Agriculture Organization of the United Nations (FAO)^2,3^, however, these datasets are not suitable to describe the spatial variation within a country^4^ in a finer scale. Particularly in the countries where landholding size is apparently small, a relatively high spatial resolution data is required to understand the variation and trend. Moreover, to analyze the impacts of climate change on crop yield long-term spatial and temporal data is required^5^. Most developing countries have a relatively poor database of location-specific weather and historical crop yield data due to associated logistics and financial costs and hence it becomes difficult to target adaptation interventions.

Remote sensing provides an opportunity to monitor vegetation over time and space. There is an increasing availability of high spatial and temporal resolution data of vegetation indices, and climatic parameters for the same areas. Models based on remote sensing typically use vegetation index (VI), with other biophysical parameters to estimate crop vigor. Gross Primary Production (GPP) is an important indicator of assimilation of terrestrial carbon (C) in the biosphere to study the global carbon cycle^6–9^. Numerous studies are conducted to estimate GPP of the global biosphere, primarily they are general, and focused on forest ecosystems as their huge potentiality to sequestrate atmospheric CO_2_^10^. The agricultural ecosystem has rather attained less focus, although it covers 15 million sq km worldwide. Though the forest ecosystem is the primary sink of atmospheric CO_2_, the managed agro-ecosystems act as a significant sink of CO_2_ in the terrestrial biosphere^11–13^ and RS-based models indicate that agricultural NPP over India accounts for 55–60 percent of national NPP^14^. Recent studies take account of carbon sequestration in agriculture to allocate values in agricultural production^15^.

Several models are developed to estimate GPP and mainly they can be grouped as (1) Enzyme kinetic (EK) models^16,17^, (2) Empirical models^18^, (3) Solar-induced chlorophyll fluorescence (SIF) models^8,19–21^ and (4) Light use efficiency (LUE) models^22,23^. LUE models are the most preferred approach for use of RS data and have undergone extensive improvements and modifications which add to their accuracy and adaptability to a wide range of conditions^24^.

MODIS primary productivity (MOD17) products are significantly used worldwide for C sequestration and biosphere modeling, however, the limitations of over and underestimation of the product can’t be ruled out because of uncertainties from various upstream inputs^25–27^. Jiang and Ryu provided an alternative set of GPP datasets, namely the breathing Earth system simulator (BESS), based on a simplified process-based model^28^. Instead of standard MODIS products, BESS is capable to serve as an independent GPP dataset^29^. Another global moderate-resolution dataset of vegetation GPP for 2000–2016 was developed by Zhang *et al*. and it showed satisfactory performance across a wide range of biome types^27^. However, computed by different algorithms (e.g. MOD17) and using different input parameters (e.g. JRA-25, NECP), the performance of these GPP products vary widely in various climate zones^30–32^. Additionally, over a complex ecosystem to improve the GPP estimation better-quality meteorological data and land-use information on a finer spatial scale are required^33^.

In this paper, we have used GPP at 500 m pixel level as a proxy to develop a dataset of integrated crop productivity for the last 20 years for India, a home base for almost 100 million smallholders with less than 2-hectare landholdings. We have further developed normalized integrated crop yield data at 500 m scale by downscaling measured historical crop yield data available at the aggregated district scale using GPP data. We expect these two data sets to help develop a better understanding of trends, and impacts of climatic variability on agriculture, and to contribute to loss estimation in the crop yields, much needed for developing improved insurance products and schemes. India has a large crop insurance scheme that is suffering due to the scarcity of disaggregated data at the village or lower scale.

## Methods

**Fig. 1:**
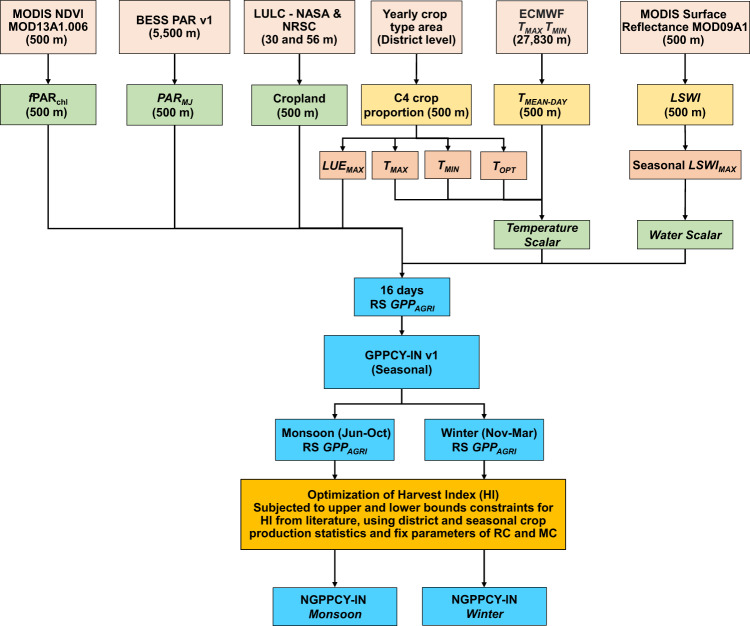
Data and procedure used to calculate seasonal primary productivity and its validation.

To calculate agricultural GPP and validation, environmental parameters and published data were used. The method consisted of four steps (1) modeling GPP yield seasonally over time and extracting cropland-related GPP, (2) summarizing agro-GPP at the district level, (3) calibration and validation, and (4) remote sensing normalized GPP (Fig. 1).

### Study area

For the present study, the GPP was calculated for India. With a total of 160 MHa of arable land, and 82 MHa of gross irrigated area, agriculture is one of the primary occupations of almost 50% of inhabitants and it contributes a significant share (16%) to the national GDP. The total food grain production was 297 MT in 2020, and the gross value added by the agriculture and allied sector was USD 276 billion in the 2020 Fiscal year.

The cropping pattern of India is highly complex and varies with climatic conditions, topography and available resources. Primary it’s a rice-wheat-maize/ millet system, with a dominancy of the rice-wheat system. In the monsoon paddy, maize, sugarcane, millets, cotton, soybean etc., and in winter wheat, gram, mustard, and barley are primarily cultivated. Based on the area under cultivation and its economic importance major C3 (castor, cotton, groundnut, pulses, pigeon pea, rice, soybean and sunflower) and C4 (finger millet, maize, pearl millet, sorghum and sugarcane) crops were considered for monsoon season. For the winter season, the following C3 crops are considered: barley, chickpea, linseed, mustard, safflower, sesamum, rice and wheat, and winter sorghum (C4). Later, these C3 and C4 crop areas were used to generate C3/C4 fraction map for each season and each year. For the present study, 557 districts of India were selected. The selection of districts was based on the availability of data at regular intervals and major crop-producing areas.

### Data used

The online and offline datasets are available in different spatial and/ or temporal resolutions. For the present study LUE model, namely, Vegetation Photosynthesis Model (VPM) is used which disaggregates vegetation canopies into two components: photosynthetically active vegetation (PAV) and non-photosynthetic active vegetation (NAV)^25^. To calculate GPP the datasets were resampled spatially and temporally to synchronize with MODIS 500 m global dataset as the primary inputs e.g., vegetation index (NDVI) and land surface water index (LSWI) in the VPM model is derived from MODIS. The details of the data used in VPM and further analysis and source are presented in Table 1.

**Table 1:** Parameters and other datasets for agricultural GPP calculation.

Parameters	Original data source	Native resolution (m)	Temporal availability
*f*PAR	MODIS/TERRA NDVI\	500 (calculated)	16 days
	(https://lpdaac.usgs.gov/products/mod13a1v006/)		
PAR	Breathing Earth System Simulator (BESS) Radiation v1^28^	5,500	Daily
T_scalar_	ERA5 Daily Aggregates - Latest Climate Reanalysis, ECMWF	27,830	Daily
	(https://cds.climate.copernicus.eu/cdsapp#!/home)		
W_scalar_	MODIS/TERRA surface reflectance	500 (calculated)	8 days
	(10.5067/MODIS/MOD09A1.006)^46^		
T_min_/T_max_/T_opt_	C3/C4 crop fraction at district level from Govt. data^50^	500 (calculated)	Seasonal
Cropland filter	Landsat^40^ and AWiFS^41^	30 and 56	One time each (2015 and 2018 respectively)

### Vegetation index

The present MODIS VI product (v06) is computed from atmospherically corrected bi-directional surface reflectance that has been corrected for water, clouds, heavy aerosols, and cloud shadows (https://lpdaac.usgs.gov/products/mod13a1v006/). The MOD13 and MYD13, are derived from MODIS TERRA and AQUA respectively to produce VI products. For the present study, MOD13 VI with 16-day intervals was used because of its availability from an earlier period than MYD13. Estimating GPP using normalized (NDVI) and enhanced (EVI) is common and researchers are testing other vegetation indices for performance evaluation. Though EVI shows better performance over high biomass conditions^34^, NDVI responds better in a wider range and is sensitive to low biomass areas^35^. Additionally, NDVI and GPP show better simultaneous responses in wet summer conditions^36^. In India majority of the croplands are arid/semiarid areas and rainfed, thus NDVI is the better choice for the present study.

Though the present method has used MODIS NDVI, a successor of MODIS such as Suomi NPP Visible Infrared Imaging Radiometer Suite (VIIRS) can be used. The VI products of VIIRS are available in Google Earth Engine as VNP13A1.

### Estimating LUE_MAX_ with C3C4 crop fraction

The C3 and C4 plants distinctly respond differently to the rising CO_2_ concentration as well as they differ from the change in solar radiation and temperature, and physiological functions^37^. In VPM model LUE_MAX_ (ε*) is a critical factor and vegetation dependent, which can be parameterized as ecosystem quantum yield based on the Michaelis-Menten light response function^35^. Earlier studies used Earth Stat global major crop type distribution, SPOT vegetation products etc. to estimate C4 vegetation fraction, however, they are either static and do not comply with changes in cropping practice over time or coarse resolution (1°). To generate C4 crop faction maps to further use in LUE, the present study used district-level data of C3C4 crops for each year for monsoon and winter seasons and resampled at 500 m resolution to integrate with MODIS NDVI products. To estimate LUE_MAX_ following equation was used and calculated for each pixel, where prop_C3_ and prop_C4_ are eddy-covariance measurements of C3 and C4 crops.

1 $${\varepsilon }^{\ast }={{\rm{LUE}}}_{{\rm{C}}3}\times {{\rm{prop}}}_{{\rm{C}}3}+{{\rm{LUE}}}_{{\rm{C}}4}\times {{\rm{prop}}}_{{\rm{C}}4}$$

where, LUE_C3_ and prop_C3,_ LUE_C4_ and prop_C4_ are maximum light use efficiency and the fraction of C3, C4 crops respectively. Primarily the LUE_C3_ and LUE_C4_ (1.37 gC MJ^−1^ APAR, and 1.64 gC MJ^−1^ APAR) were taken from literature^35,38^ and post-optimization the values of LUE_C3_ and LUE_C4_ were set to 1.388 and 1.542 gC MJ^−1^ APAR (this study).

### Climatic variables

The daily photosynthetically active radiation or PAR (W m^−2^) was obtained from BESS datasets (Breathing Earth System Simulator (BESS) Radiation v1^28^ and synced with the model simulation interval of 16 days. For daily daytime mean temperature, daily maximum and minimum air temperature at 2 meters were obtained from ERA5-Climate Reanalysis (https://cds.climate.copernicus.eu/cdsapp#!/home) was used and synced with model interval. Both the datasets are 27,830 m resolution and it was upscaled to 500 m to match with MODIS NDVI data.

### Cropland mask

Among other off-the-shelf products, MODIS LULC (MOD12) is commonly used by the researcher community because of its uninterrupted availability of data at high spatial (500 m) and temporal resolution (yearly). Though these datasets provide satisfactory estimations in global to country-level study, for a country like India where the majority of the agricultural land holdings are less than 1 Ha, is not the best choice^39^. For the present study Global Food Security-Support Analysis Data^40^ was used to extract cropland areas at 30 m resolution from the calculated GPP data. As observed from longtime cropland statistics published by Govt. of India, the temporal variation in the cropped area at the district level is insignificant and agrees with the GSFAD crop mask (data not shown) thus it was further used. Furthermore, this annual cropland data was rectified by National Remote Sensing Center (NRSC)^41^ seasonal crop mask (56 m) data (2018) and crop masks were created for both monsoon and winter seasons.

Seasonal cropland masks based on the phenology for each year would be preferable to compare modeled agricultural GPP with crop statistics data. However, the purpose of the study is to use a simplified and replicable method to increase operational feasibility, and for that, a static cropland mask was used.

### Process flow in the VPM model

The VPM model exploits the fact that photosynthetic active vegetation or chlorophyll (fPAR_chl_), which is contributed by the vegetation canopies into photosynthetically active vegetation (PAV) and non-photosynthetic active vegetation (NPV)^25^. The GPP is the product of LUE, fPAR and PAR (Eq. 2). For fPAR, the method developed by Myneni and Williams^42^ was used (Eq. 3) where a = 1.24 and b = 0.168^43^. This method is based on the radiation transfer model and is reckoned to be robust in vegetation clustering, variance in pixels, leaf orientation and optical properties. Estimating LUE (ε) from LUE_MAX_ (ε^*^) requires downregulating by temperature (*T*_*scalar*_), water (*W*_*scalar*_) and phenology (*P*_*scalars*_) scalars (Eq. 4). *P*_*scalar*_ was set to 1 because of leaf emergency in dominant crops such as rice/wheat among the other crops considered here during rainy and winter seasons^25^. In Eq. 5, *T*, *T*_*max*_, *T*_*min*_ and *T*_*opt*_ are daytime mean temperatures followed by maximum, minimum and optimum temperatures for the photosynthetic activity of each typical vegetation type. For C3 and C4 crops the maximum, minimum and optimum temperatures (C) are 40, 5 and 25^44^; 42, 8 and 30^38,45^ respectively. For water scalar (Eq. 7) LSWI is used (Eq. 6) which is sensitive to canopy water stress and a product of NIR (841–876 nm) and SWIR (1628–1652 nm) bands of MODIS surface reflectance (10.5067/MODIS/MOD09A1.006)^46^.

2 $${\rm{GPP}}=\varepsilon \times {\rm{fPAR}}\times {\rm{PAR}}$$

3 $${\rm{fPAR}}={\rm{a}}\times {\rm{NDVI}}-{\rm{b}}$$

4 $$\varepsilon ={\varepsilon }^{\ast }\times {T}_{{\rm{scalar}}}\times {W}_{{\rm{scalar}}}\times {P}_{scalar}$$

5 $${T}_{scalar}=\frac{\left(T-{T}_{max}\right)\times \left(T-{T}_{min}\right)}{\left(T-{T}_{max}\right)\times \left(T-{T}_{min}\right)-{\left(T-{T}_{opt}\right)}^{2}}$$

6 $$LSWI=\frac{NIR-SWIR}{NIR+SWIR}$$

7 $${W}_{scalar}=\frac{1+LSWI}{1+LSW{I}_{max}}$$

Post GPP simulation for every 16 days, it was summed for the whole season and seasonal crop masks were used to nullify non-agricultural pixels. This simulation process was performed in Google Earth Engine cloud computing facilities. The final seasonal GPP product for two decades was calibrated and validated with yearly Govt. crop statistics data and named as GPPCY-IN. Since the crop production statistics data published at the district level and yield loss calculation required more spatially explicit input, the crop yield data was further normalized by the fraction of actual GPP_RS_ and GPP_RS-Mean_ at pixel level and additional dataset (NGPPCY-IN prepared for 2001–2017. To calculate NGPPCY-IN, we have used fraction of GPPCY-IN at pixel level and mean of pixels of a district, further it was multiplied by the crop-combined primary productivity that was derived from the crop statistics. Reported in T/Ha this dataset (NGPPCY-IN) translates the district level primary productivity at pixel level.

## Data Records

**Fig. 2:**
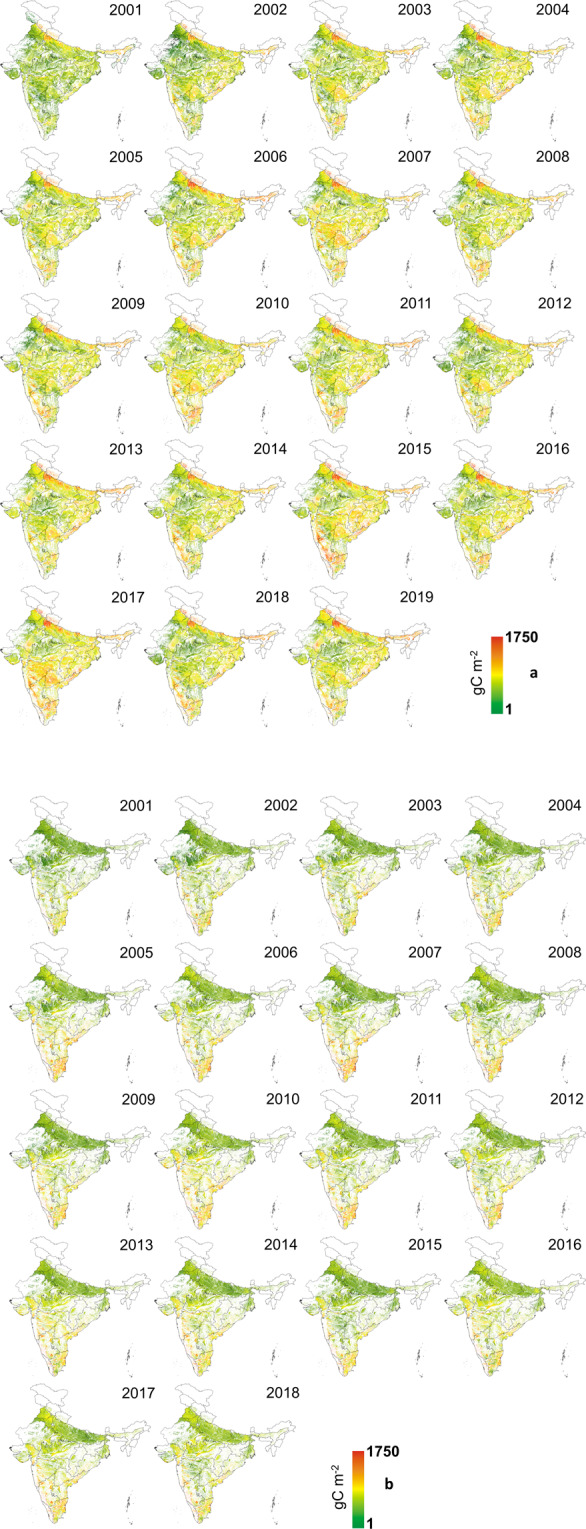
GPPCY-IN over India in the monsoon season (June to October), 2001–2019 (2a) and winter season (November to March), 2001–2018 (2b).

The GPPCY-IN product is available at 16 days intervals at 500 m resolution. However, we have integrated it seasonally from 2001 to 2019 to compare with ground data (till 2017) for the monsoon (Fig. 2a) and winter (Fig. 2b) seasons. Additionally, the NGPPCY-IN is also prepared for 2002–2017 (not shown here). These datasets are available with a longitude-latitude projection under the WGS84 datum. On request, the calibration coefficients, and radiation use efficiency correction factors will be made available. Both the datasets (GPPCY-IN^47^ and NGPPCY-IN^47^) are uploaded on ‘*figshare*’. In the repository, the filenames, e.g. GPPCY-IN-M2001or NGPPCY-IN-W2002 represent ‘agricultural GPP of India - Monsoon - 2001’ and ‘normalized GPP of India - Winter – 2002’ respectively.

## Technical Validation

Even after taking standard precautions, satellite observations are needed to be validated with ground observations to minimize errors. In the present study district-level crop statistics data from 2001 to 2017 for monsoon (n = 13) and winter crops (n = 11). The selected crops cover more than 90% sown area. From crop statistics datasets and using literature-based values for harvest index (HI), respiration coefficient (RC) and moisture content (MC) seasonal GPP was estimated.

These parameters differ based on the crop, location, and season; and hence a single value cannot be representing all cases. Here, literature-based constant values for RC and MC and the upper and lower bounds for HI were used. Hereafter, to obtain realistic coefficient values an optimization problem was set wherein the difference in GPP estimates from satellite and production data was minimized by finding optimal HI coefficient values for each crop, season and year. The obtained HI coefficients were then further tested against *consecutive* years’ data for independent validation. The optimization process is explained below with details on the calibration and validation process.

### Optimization process

The objective function (Z) minimizes the difference between satellite estimates of GPP and GPP estimated from crop production datasets by setting the values of the optimal coefficient for harvest index which varies for each district, crop, season and year.

8 $$Z=Sat.GP{P}_{D}1.15\ast {\sum }_{i=1}^{n}\left(\frac{PRO{D}_{i}}{H{I}_{i}}\ast \left(1+R{C}_{i}\right)\ast \left(1-M{C}_{i}\right)\right)$$

where Z is the objective function that minimizes the difference between satellite estimated GPP and the GPP estimated from crop production statistics. This objective function was executed for each district independently for each crop season and year.

Sat.GPP is the satellite estimated GPP over the cropland.

PROD is crop production of i^th^ crop. This production accounts for the litterfall and energy efficiency factors of each crop.

HI, RC and MC represent the harvest index, respiratory coefficient and moisture content, respectively for i^th^ crop.

The constrained optimization was performed using MATLAB^©^’s *fmincon* function. The function *fmincon* finds a constrained minimum of a scalar function of several variables starting at an initial estimate. A set of lower and upper bounds was defined on the design variables so that the solution is always in the range. The lower and upper bounds on the harvest index were set for each crop and each district. The midpoints of lower and upper bounds were taken as starting points in the optimization.

### Calibration and validation

Optimal coefficient values for HI, RC, MC and radiation use efficiency were obtained for each year in the calibration run. During the validation, these coefficients were then applied to the consecutive year’s data of satellite GPP and crop production. The coefficient values obtained in the calibration run for a given year were used in the validation run for the next year.

Although there could be several ways of doing calibration and validation, we opted for validation based on the above simpler methodology. In the absence of crop statistics (mainly cropped areas) in the near-real-time, the previous year’s coefficients are a closer approximation since the cropped areas, cultivar/management options will not have drastic changes. The above method referenced over the previous cropping season remains simpler from the practical applicability point of view. However, there are significant variations observed year to year in the weather patterns and in such conditions, the assumptions based on climate analogs either in space or in time could have been better approximations. Nevertheless, it would increase the complexity of the process and limit its applicability. In the above-mentioned process, the validation run does not have any optimization. The flow chart of the calibration and validation process is shown in Fig. 1.

**Fig. 3:**
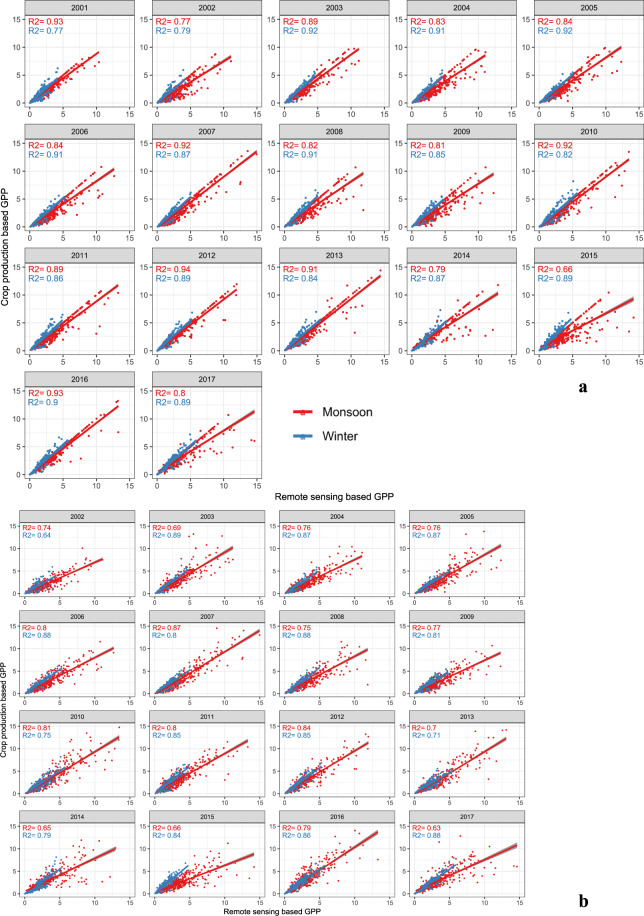
Calibration (3a) and validation (3b) of GPPCY-IN with crop production database of monsoon and winter seasons.

**Fig. 4:**
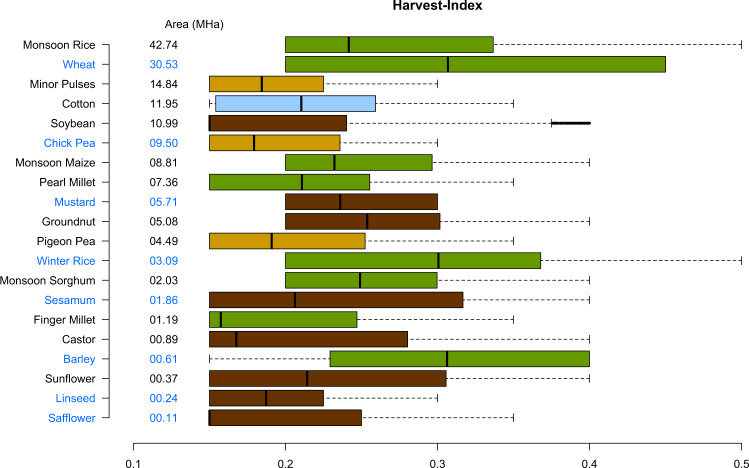
Harvest Index of selected crops for monsoon (in black) and winter season (in blue). The average value of HI for sugarcane was 0.69 and not shown in Fig. 4 due to scale difference. The values are arranged in decreasing order of average area from 2015–17. The green, brown and yellow colors represent cereals, pulses and oilseeds.

The estimated GPP from ground-level govt. data for each year was also compared with calibrated and validated (for the consecutive years) GPP at the district level (Fig. 3a,b). As the govt. has published crop statistics data till 2017, the calibration and validation process were confined to this year. In the monsoon season, the coefficient of determination (R^2^) between remote sensing estimated GPP was close with calibrated datasets (R^2^ = 0.86) and it was strongly agreed with next year’s ground estimated GPP during the validation (R^2^ = 0.77). For the winter season, the coefficient of determination between remote sensing and ground estimated GPP was 0.87 and 0.83 for calibration and validation respectively. The model optimized values of the HI and corresponding average crop area in India are presented in the boxplot in Fig. 4 (except sugarcane). The mean values of optimized HI for different crops varied from 0.15 to 0.5; monsoon rice, winter rice and wheat had higher average HI of 0.24, 0.30 and 0.32, respectively compared to other crops. The average value of HI for sugarcane was taken 0.69 in all areas.

In general, the dataset had better calibration and validation during the monsoon season than in the winter season. In the winter season, a large area in the rainfed agricultural areas stays fallow with sparse cultivation of minor local crops which are not often reported. Additionally, districts with dense tree cover with intermediate patches of croplands were also reported with high GPP, though agricultural GPP is less as per crop production statistics. To avoid the discrepancies and to improve the correlation between modeled and observed GPP, districts with less than 75,000 Ha cropped areas were excluded along with the districts that have less than 30% area (of considered crops) of the total cropped area. These excluded districts account for 4% and 11% of the mean national cropped area in monsoon and winter seasons.

The normalized root mean square (nRMSE) values of monsoon seasons vary from 13% to 29% with an average of 20% for calibration years and validation years the nRMSE range is 24% to 36% with an average of 28%. For the winter season the mean nRMSE are 20% and 28% for calibration years and validation years respectively.

## Usage Notes

**Fig. 5:**
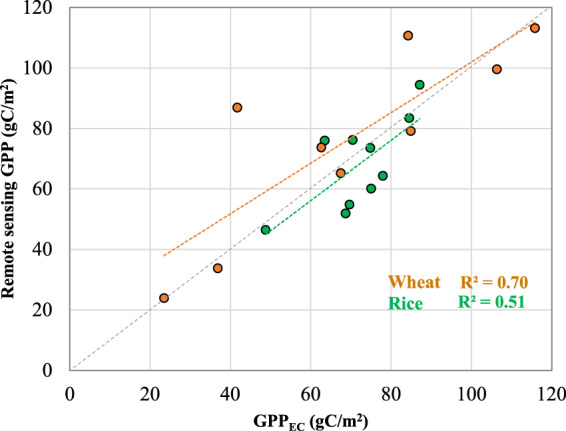
Validation of cumulative 16 days estimated GPPCY-IN with measured GPP by eddy covariance approach (GPP_EC_) in winter wheat and dry season rice.

The calculated datasets, GPPCY-IN and NGPPCY-IN are a valuable resource to monitor crop yield trends, estimate yield loss and understand the interrelationship between climate variability and extreme climatic events. The GPPCY-IN is available for each cropping season and can be used to estimate crop status. Once further crop production statistics datasets will be available, these datasets will be updated in the due course. The derived datasets show that the calculated GPP is capable to capture the seasonal variations in reference to GPP_EC_ (Flux tower-based measurements). The open-source EC data (such as FLUXNET) primarily focused on forest/ natural vegetation, for the present study, two studies were taken as a reference that is used for dry season rice^48^ and winter wheat^49^. The observed GPP_EC_ in rice and wheat were aggregated and synced with MODIS interval as in this study and found to be correlated with calculated GPPCY-IN (Fig. 5).

The developed datasets show a steady increase in agro-GPP over time which is well agreed with yearly crop production statistics data. In India, the release of yearly crop statistics is usually not available immediately at the end of the season, this first dataset (GPPCY-IN) could be an alternative data source to estimate integrated crop yield along with trends in crop production. The second product, NGPPCY-IN will serve as a proxy for crop yield statistics at the pixel level. NGPPCY-IN translates aggregated district-level crop statistics at the pixel level (ca 25 Ha), which would be a valuable resource to estimate the yield loss at comparatively higher resolution. Additionally, the payouts of crop insurance schemes are often delayed primarily because crop cutting for yield estimation a labor-intensive process. The developed datasets show GPP can be used as a proxy for integrated crop yield and limiting the data collection errors on the ground together with boosting the process of loss estimation. However, for operational purpose the limitations of remote sensing based observations further need to be calibrated with precise ground observations, such as crop-cutting experiment (CCE) in few sentinel sites.

**Fig. 6:**
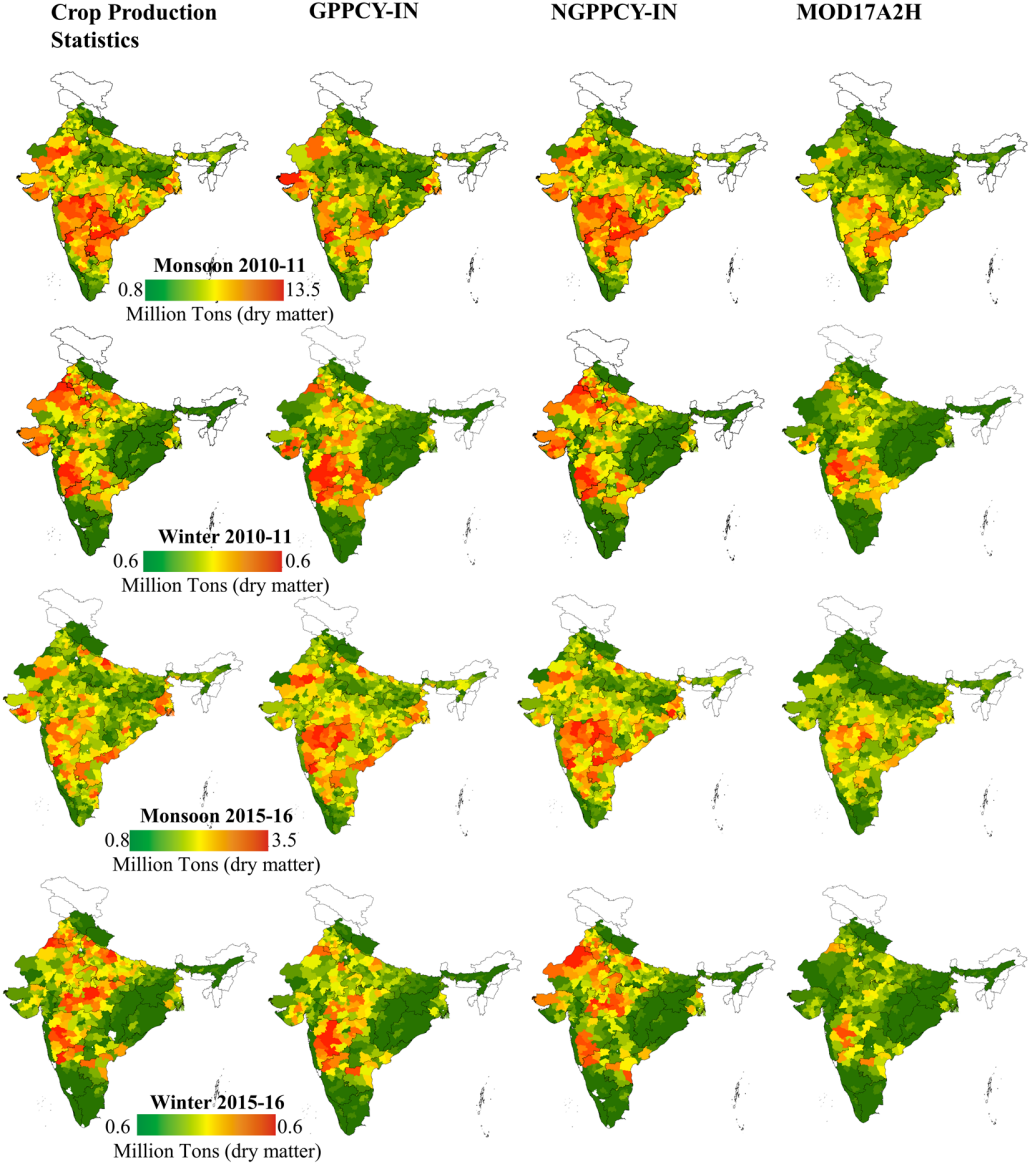
Comparison of GPPCY-IN with crop production statistics, NGPPCY-IN and MOD17 datasets in La Nina strong (2010–11) and El Nino strong (2015–16) years. These datasets are aggregated at district level.

**Fig. 7:**
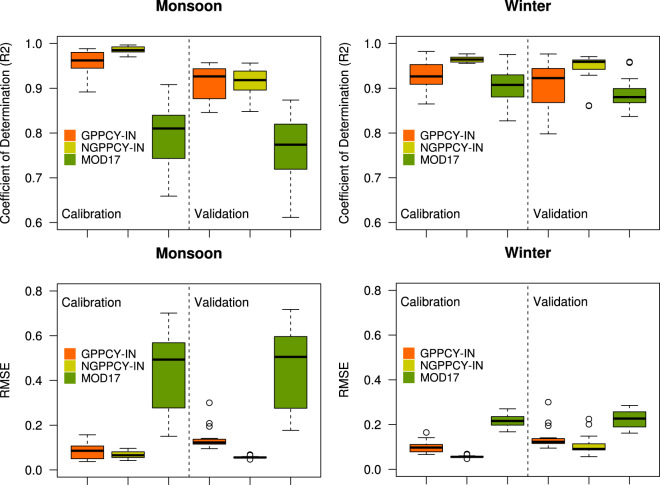
Boxplot showing distribution of calibrated and validated GPPCY-IN, NGPPCY-IN (present study) and MOD17 compared to crop production statistics in Indo-Gangetic Plain.

Compared to the other global products such as MOD17A2H, GPPCY-IN shows a better correlation with observed data (Fig. 6). Calculated at the pixel level and aggregated at the district level, the GPPCY-IN shows a higher correlation compared to MOD17A2H. Anomalous behaviors have been observed in the Indo-Gangetic plain for two specific years (2010–11 and 2015–16), the variation is up to 30% (underestimate) at the national level with GPPCY-IN. Particularly in the Indo-Gangetic plain, which consists of 5 states of India with approx. 30MHa cropland and produces 50% of the considered crops, GPPCY-IN and NGPPCY-IN show a better correlation with reported crop production statistics than MOD17A2H (Fig. 7).

Users of the products should be aware of assumptions in developing GPP products that could limit its applications. The cropping pattern in India is extremely complex and the sowing/harvesting time varies over the country and significant adoption of agroforestry can happen in local areas. The present study considered November to March as winter crop season, however, in east India this might extend till April. The exclusion of minor crops that locally make a significant difference in a few districts, extended duration of crops beyond dominant cropping seasons like cotton and sugarcane and lumping of coefficients across the region. Additionally, these seasonal GPP datasets are calculated seasonally (June to October and November to March) that exclude the months of April and May, hence the aggregation of seasonal GPP would not represent annual GPP. The primary reason behind excluding these two months is, that in India the primary crop seasons are Monsoon (June to October) and Winter (November to March) and the measured crop statistics is available accordingly.

## Data Availability

GPPCY-IN was developed on Google Earth Engine (JavaScript) and calibration/validation was performed in MATLAB in the local system. A code for GPPCY-IN calculation can be found here: https://github.com/Prasun-G/GEE-code-for-Agricultural-GPP-India.
